# TFEB: A Emerging Regulator in Lipid Homeostasis for Atherosclerosis

**DOI:** 10.3389/fphys.2021.639920

**Published:** 2021-02-17

**Authors:** Manman Li, Zitong Wang, Pengyu Wang, Hong Li, Liming Yang

**Affiliations:** ^1^Department of Pathophysiology, School of Basic Medical Sciences, Harbin Medical University, Harbin, China; ^2^Department of Pathophysiology, Harbin Medical University-Daqing, Daqing, China

**Keywords:** TFEB, lipid homeostasis, atherosclerosis, post-translational modifications, lipid transporters, lipophagy, lipid mediators

## Abstract

Atherosclerosis, predominantly characterized by the disturbance of lipid homeostasis, has become the main causation of various cardiovascular diseases. Therefore, there is an urgent requirement to explore efficacious targets that act as lipid modulators for atherosclerosis. Transcription factor EB (TFEB), whose activity depends on post-translational modifications, such as phosphorylation, acetylation, SUMOylation, ubiquitination, etc., is significant for normal cell physiology. Recently, increasing evidence implicates a role of TFEB in lipid homeostasis, *via* its functionality of promoting lipid degradation and efflux through mediating lipophagy, lipolysis, and lipid metabolism-related genes. Furthermore, a regulatory effect on lipid transporters and lipid mediators by TFEB is emerging. Notably, TFEB makes a possible therapeutic target of atherosclerosis by regulating lipid metabolism. This review recapitulates the update and current advances on TFEB mediating lipid metabolism to focus on two intracellular activities: a) how cells perceive external stimuli and initiate transcription programs to modulate TFEB function, and b) how TFEB restores lipid homeostasis in the atherosclerotic process. In-depth research is warranted to develop potent agents against TFEB to alleviate or reverse the progression of atherosclerosis.

## Introduction

Cardiovascular diseases cause serious harm to human life and health (Mialet-Perez and Vindis, 2017). Atherosclerosis, characterized by lipid accumulation and inflammatory cell infiltration of the arterial wall, is the main pathological basis of cardiovascular diseases (Libby et al., 2011; Herrington et al., 2016). Upregulated lipid uptake and synthesis as well as deregulated degradation and transport make atherosclerotic cardiovascular events appear (Weber and Noels, 2011). Lipid homeostasis is controlled by the coordinated regulation of various metabolic pathways involving *de novo* synthesis, absorption, storage, transport, and breakdown of lipids (Yuan et al., 2012). Disruption of this balance may lead to lipid accumulation, which in turn gives rise to a multiplicity of life-threatening metabolism-related diseases currently, such as cardiovascular diseases, fatty liver, diabetes (Luo et al., 2020). The ways of regulating lipid metabolism are tightly interlinked and intricately entwined. Multiple lines of evidence have established that making the appropriate adjustments to each link from lipid synthesis to degradation and reuse, paying attention to the key target pathways in which they participate, is crucial to lipid homeostasis (Thelen and Zoncu, 2017).

**FIGURE 1:**
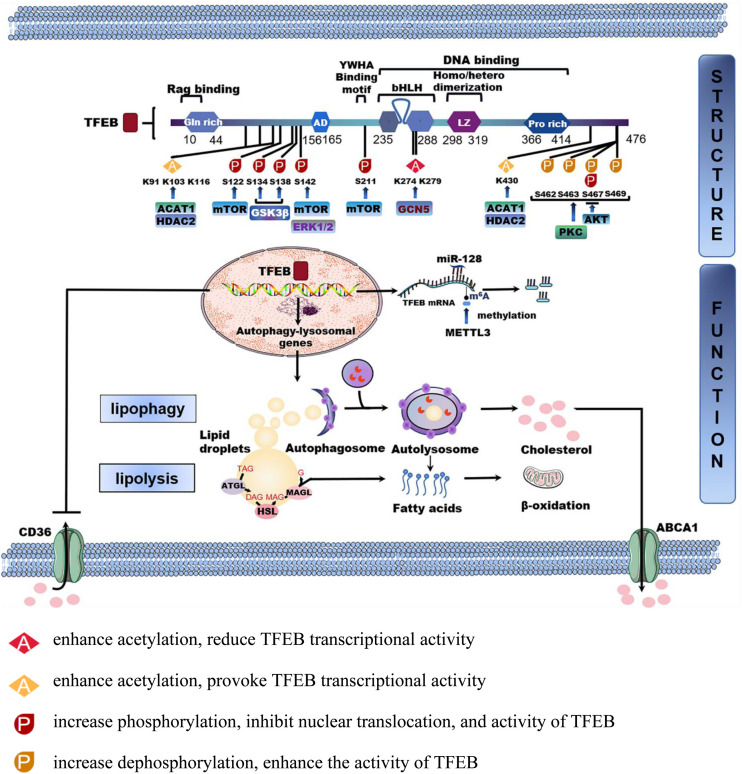
The structure and function of TFEB in lipid homeostasis. TFEB is a protein consisting of 476 amino acid residues, mainly including glutamine-rich (GIn rich), an acidic transcription activation domain (AD), basic helix-loop-helix leucine-zipper (bHLHZIP) structure, and proline-rich motifs (Pro rich). Phosphorylation and acetylation are the most important post-translational modifications to regulate TFEB activity. The phosphorylated or acetylated TFEB sites by different proteins are shown in the figure. TFEB is also regulated by transcription and non-coding RNA. MicroRNA-128 targets TFEB to tune the transcription of autophagy-related genes. Methyltransferase like 3 (METTL3) inhibits the transcriptional activity of TFEB by methylating two m^6^A residues. After being activated into the nucleus, TFEB regulates downstream autophagy-lysosomal genes to modulate lipophagy and neutral lipolysis. Furthermore, TFEB also regulates lipid transport processes such as lipid uptake and cholesterol efflux by CD36 and ATP-binding cassette transporter A1(ABCA1).

As a salient transcription factor belonging to the microphthalmia/transcription factor E (MiT/TFE) family, transcription factor EB (TFEB) has been identified to modulate basic intracellular homeostasis (Napolitano and Ballabio, 2016). The activity and function of TFEB are also governed by a quite complex but only partially defined regulatory network consistent with other proteins, including transcription, translation, and the most common post-translational modification. During nutritional deficiency, TFEB translocates into the nucleus combined with downstream target promoters to regulate cell physiological processes, which not only takes part in ontogeny and angiogenesis but also has arisen as a master regulator of autophagy and lysosome biosynthesis (Xu and Ren, 2015; Fan et al., 2018). Evidence has mounted that TFEB is involved in the regulation of lipid metabolism in many diseases. For example, TFEB activates autophagy to indirectly inhibit liver steatosis and against weight gain in obesity (Zhang Y. et al., 2018). TFEB also contributes to being a potential therapeutic target for non-alcoholic fatty liver disease *via* the beneficial effect in regulating lipolysis and lipophagy (Yu et al., 2020). Remarkably, it has been discovered in recent years that TFEB also participates in lipid metabolism through regulating lipid mobilization and oxidation, inhibiting lipid uptake, promoting intracellular lipid degradation and efflux, preventing lipid accumulation by coordinating autophagy-lysosomes and modifying lipid transporters and lipid mediators, thus alleviating the progression of atherosclerosis and stabilizing plaques (Chen L. et al., 2017; Figure 1).

Despite the considerable amount of work being conducted to understand the role of TFEB in cell metabolism and lipid homeostasis, several instances of its involvement in the regulation of lipid levels in atherosclerosis and related diseases have only recently been identified. Given the consummated investigations and the unmet challenges, this article focuses on the current cognizance of the several ways to activate TFEB and the integrated network mechanism of TFEB linking lipid homeostasis with atherosclerosis. In addition, we delineate some TFEB activators as potential therapeutic candidates in mitigating the pathological progression of atherosclerosis.

## Basic Structure and Function of TFEB

Transcription factor EB, consisting of 476 amino acid residues, is a member of MiT/TFE family with high sequence homology whose components include (i) the basic helix-loop-helix leucine-zipper (bHLHZIP) structure, (ii) an acidic transcription activation domain, (iii) glutamic acid-rich domain, (iv) serine-rich domain and other domains (Fisher et al., 1991). Not only can TFEB recognize palindromic CACGTG E-box, but also asymmetric TCATGTG M-box sequence that is unavailable to other bHLHZIP transcription factors (LeBlanc et al., 1998). Moreover, TFEB combines with homologous or heterologous deoxyribonucleic acid (DNA) groups in the form of dimers to initiate transcription of the corresponding gene (Brady et al., 2018a). For instance, the effect of the autophagy process is effectively regulated by TFEB, as it promotes autophagy formation, autolysosome fusion, and degradation through binding to the promoter regions of autophagy-related genes (Shin H. R. et al., 2016). Intriguingly, recent evidence strongly suggests that TFEB overexpression accelerates the degradation of a large number of autophagy substrates such as proteins, lipid droplets, damaged mitochondria, and endoplasmic reticulum, indicating the non-negligible role of TFEB in selective organelle autophagy (Wang et al., 2020; Yao et al., 2020).

Transcription factor EB is also known to be a vital regulator of lysosomal biosynthesis-related genes by connecting with the coordinated lysosomal expression and regulation (CLEAR) motif in the promoter region responsible for lysosome regeneration and biogenesis (Curnock et al., 2019). Another function of TFEB on lysosomes is to induce lysosomal exocytosis, a process in which lysosomes fuse with the plasma membrane, followed by secretion of their contents outside cells (Di Paola and Medina, 2019b). TFEB also exerts emerging effects on lipid metabolism by way of directly or indirectly modulating lipid uptake, transport, and degradation. To realize the above functions, cells need to start a TFEB transcription program in response to various kinds of environmental cues. Therefore, it is important to elucidate how the upstream factors regulate TFEB activity with subsequent enhancement of autophagy and lipid metabolism.

## The Specific Mechanism for TFEB Activation

When cells are stressed by factors such as starvation, lysosomal stress, and mitochondrial damage, they must respond quickly by regulating the rapid shift of cytoplasmic TFEB to the nucleus for binding to DNA elements and activating the transcription of target genes, so as to adapt to the environment and restore their dynamic balance (Willett et al., 2017). However, the network of mechanisms regulating TFEB activity and function is complex and still not fully defined. At present, there is strong evidence that TFEB is strictly regulated by transcription, translation, post-translational modification, and protein-protein interaction.

### Transcriptional Level, Non-coding RNA, and Translation

Transcription factor EB is regulated by different factors at the transcription level. The key stage of gene expression is the initiation of transcription, whose switch is the promoter, a segment of DNA sequence specifically recognized and bound with the ribonucleic acid polymerase. Most promoters are located upstream of the transcriptional starting point of structural genes, controlling the initiation time and the degree of expression to determine the activity of target genes. Some scholars have found that the occurrence of chromosomal translocation results from the fusion of the open reading frame of TFEB on chromosome 6 to the 5′ regulatory region of the non-protein encoding alpha gene on chromosome 11. Consequently, the TFEB promoter is replaced, contributing to the significant up-regulation of the TFEB transcription level and the expression of TFEB protein (Argani et al., 2005, 2016; Zhan et al., 2018). Forkhead box O1, a transcription factor that controls mitochondrial function and morphology, could interact with TFEB, drastically increasing the transcription and protein levels of TFEB through directly binding to its promoter (Liu et al., 2016). Non-receptor kinase Janus kinase 2 (JAK2) knockdown alleviates TFEB promoter activity, expression, and nuclear localization, subsequently blocking autophagy and lysosome function. The observations of *in silico* analysis and chromatin immunoprecipitation (ChIP) assays highlight that activating JAK2 encourages the binding of downstream transcription activator 1 and TFEB promoter, thus increasing the transcription activity of TFEB, achieving the purpose of reversing lysosomal dysfunction and restoring albumin permselectivity in glomerular disease (Alghamdi et al., 2017). Current reports showed that Krüppel-like factor 2 upregulated TFEB expression and promoter activity to exert anti-inflammatory effects in endothelial cells and diabetic mice (Song W. et al., 2019). In a recent study involving analysis by ChIP of primary hepatocytes and whole livers *in vivo* and vitro, hepatic spliced X-box binding protein 1 was found to enhance TFEB transcription and autophagy by occupying the 743 to 523 site of the promoter of TFEB (Zhang et al., 2020).

In addition to the regulation of promoter activity, recent studies have found that microRNA and long non-coding RNA (LncRNA) are involved in regulating the transcription of TFEB. TFEB is the target of microRNA-128 in concomitantly tuning the transcription of autophagy-related genes (Settembre et al., 2011; Tiribuzi et al., 2014). The process of renal cancer caused by high expression of TEFB is related to LncRNA fusion (Malouf et al., 2015; Xia et al., 2015; Pei et al., 2019). Further evidence also links RNA methylation with TFEB regulation. Methyltransferase like 3 (METTL3) reverses the transcriptional activity of TFEB by binding to the 3’-UTR end of TFEB and concomitantly methylating two m^6^A residues, thus playing a protective role in ischemic heart disease (Song H. et al., 2019).

A novel modulations of TFEB activity at the translation level has emerged, in which TFEB translation is suppressed by both of MA3 domains within programmed cell death 4, which is a tumor suppressor propitious to lysosome dysfunction related diseases (Chen et al., 2020). Interestingly, salt-inducible kinase 2 (Negoita et al., 2019) and spermidine (Zhang et al., 2019) may affect the expression of TFEB in cells from the perspective of translation. Among the studies on the activity of TFEB, the most concerned is the modification of post-translational proteins, including acetylation, phosphorylation, SUMOylation, and ubiquitination.

### Post-translational Modification

#### Phosphorylation and Dephosphorylation

Our current understanding of TFEB biology posits when cells are under stress, TFEB protein activity, and subcellular localization or its nuclear-cytoplasmic shuttling are driven by phosphorylation modification mainly. The basilic phosphorylation sites include S142, S211, and serine-rich sequences at the end. S122, S134, S138, S462, S463, S467, and S469 phosphorylation also regulates the nuclear translocation of TFEB.

Mammalian target of rapamycin complex 1 (mTORC1) is currently the most thoroughly studied kinases related to TFEB phosphorylation (Nnah et al., 2019). Studies have shown mTORC1 mediates the phosphorylation at the C-terminal S211 amino acid of TFEB (Martina et al., 2012). When nutrients are rich and energy is sufficient, vacuolar H^+^-AT-Pase forms a complex interacting with Rag GTPase and other factors. 14-3-3 is a cytoplasmic chaperone protein that keeps TFEB isolated in the cytosol, which masks the nucleus entry signal after binding to TFEB, resulting in the loss of TFEB activity temporarily (Xu et al., 2019). The activated Rags recruit mTORC1 to the lysosome membrane through binding to the mTORC1 component raptor. At this time, the activated mTORC1 phosphorylates the S211 site of TFEB, which promotes 14-3-3 binding sites (Roczniak-Ferguson et al., 2012). Experiments with the above mechanism have proved that, due to rich nutrition, mTORC1 is increased to promote the phosphorylation of TFEB S211, which enhances hepatic steatosis and liver injury in high-fat diet-fed mice. Importantly, studies on hepatic biopsies of patients with non-alcoholic fatty liver disease revealed that the lower the activity of TFEB, the more serious the liver steatosis and lipid aggregation (Zhang H. et al., 2018). Conversely, when the nutrition is deprived, due to the inactivation of the RagGTP enzyme, mTORC1 dissociates from the surface of the lysosome and loses its activity, leading to the dissociation of the TFEB/14-3-3 complex. The rapid dephosphorylation of TFEB is transferred from the cytoplasm to the nucleus, thereby performing transcription function and activating downstream genes to maintain effective homeostasis.

Several other protein kinases have been found to affect the phosphorylation of TFEB. Adenosine monophosphate-activated protein kinase (AMPK), a central regulator of energy homeostasis, is recently shown to attenuate lipid accumulation and inflammation by promoting dephosphorylation and nuclear localization of TFEB independent of mTOR activity (Kim S. H. et al., 2017; Collodet et al., 2019). Extracellular signal-regulated kinase (ERK) phosphorylates the site of TFEB S142 and makes it relocated in the cytoplasm (Yonekawa et al., 2015; Li et al., 2019). Phosphorylation of TFEB S142 by mTOR/ERK inhibits nuclear translocation of TFEB in hepatocytes and thus promotes hepatic cholesterol accumulation (Wang et al., 2020a). Whereas AKT (protein kinase B) negatively controls TFEB through phosphorylation at S467 (Palmieri et al., 2017). The inactivation of glycogen synthase kinase (GSK)-3β activates TFEB by dephosphorylation and nuclear translocation of TFEB (Li et al., 2018; Theeuwes et al., 2020). GSK-3β coordinates with mTORC1 by phosphorylating TFEB S134 and S138 sites increasing the phosphorylation of S211A with the resultant acceleration of the binding of TFEB to 14-3-3 protein and eventually inhibiting TFEB’s nuclear transport. However, protein kinase C (PKC) might increase dephosphorylation and activation of TFEB by inactivating GSK3β. It is likely that PKC activators may be used as an effective treatment for lysosome-related disorders by activating TFEB to accelerate the degradation of accumulated lipid droplets (Li Y. et al., 2016). Besides, in the C-terminus of TFEB, multiple serine residues (i.e., S462, S463, S467, and S469) are phosphorylated by PKCβ for changing the TFEB protein stability instead of influencing subcellular localization (Ferron et al., 2013). As reported, TFEB is dephosphorylated by calcineurin. During starvation and physical exercise, calcineurin induction triggers the TFEB nuclear translocation synergistically with mTORC1 inhibition (Medina et al., 2015; Di Paola and Medina, 2019a; Pan et al., 2020).

#### Acetylation and Deacetylation

One of the modes of protein modification is acetylation modification, a process by which acetyl groups transfer to amino acid side chain groups (Yang et al., 2020). Researchers have found that acetylation modification of TFEB also affects its activity and localization. Histone acetyltransferase general control non-repressed protein 5 (GCN5) acetylates TFEB pointedly at K274 and K279. This results in the dimerization of TFEB being disrupted and the TFEB transcriptional activity reduced, accompanied by decreasing the biogenesis of autophagy and lysosome-mediated lipid hydrolysis (Scott et al., 2014; Wang et al., 2020b). On the contrary, cytosolic deacetylase histone deacetylase 6 (HDAC6) inhibition enhances the acetylation of TFEB and the TFEB nuclear localization initiating expression of downstream genes. However, the deacetylation site on TFEB and exact mechanisms remain unanswered (Brijmohan et al., 2018). Likewise, suberoylanilide hydroxamic acid (SAHA), a histone deacetylase inhibitor that is observed to activate the lysosomal function in human cancer cells. These results disclose a novel form that SAHA provokes TFEB transcriptional activity by recruiting acetyl-coenzyme A acetyltransferase 1 (ACAT1) and histone deacetylase 2 (HDAC2) to TFEB and enhancing TFEB acetylation on the regions containing K91, K103, K116, and K430 (Zhang J. et al., 2018). There is also a report for the inhibitory role of TFEB acetylation at K116 in microglia lysosomal biosynthesis (Bao et al., 2016). The discrepancy about whether acetylation is capable of boosting or constraining the transcriptional activity of TFEB may be due to different research backgrounds including cell types and the nature of the stimuli used. Hence, it remains to be further investigated what the pronounced functionality of TFEB acetylation is in controlling its transcriptional activity. Besides, after being deacetylated into the nucleus, TFEB needs to be appropriately modified in the nucleus before functioning, and it is different from the dephosphorylation as mentioned above.

#### SUMOylation

Transcription factor EB is also modified by the small ubiquitin-like modifier (SUMO). SUMOylation does not promote protein degradation like ubiquitination, but strengthens protein stability, regulates protein interaction and positioning, affects protein transcription activity, which involves regulating protein functions and cell life activities. The research has mapped that SUMO1 performs SUMOylation on a lysine site of TFEB protein with attenuated transcriptional activity (Miller et al., 2005).

#### Ubiquitination

Ubiquitination, a post-translational modifier, was discovered to play a part in the TFEB function. As a chaperone-dependent E3 ubiquitin ligase, STIP1 homology and U-Box containing protein 1 interacts with phosphorylated TFEB preferentially and initiate TFEB degradation through a ubiquitin-proteasome pathway. Correspondingly, non-phosphorylated TFEB exposures the nuclear localization signal, which exerts its transcriptional activity to promote the expression of the autophagy–lysosomal genes (Sha et al., 2017).

#### Others

In addition to the above post-translational modifications, methylation and protein glycosylation are also common post-translational modifications, which are connected with TFEB. Methylation includes DNA methylation, RNA methylation, or protein methylation. There are research reports showing that TFEB may regulate hypo-methylated genes in type 2 diabetes (Liu et al., 2015). It has been mentioned previously that TFEB undergoes RNA methylation *via* the m^6^A modification of its mRNA adenylate catalyzed by METTL3 (Song H. et al., 2019). Besides, TFEB activity is also regulated by a protein methyltransferase. Research has shown that under nutrient starvation, co-activator-associated arginine methyltransferase 1 (CARM1) and TFEB exhibit mutual combination and subsequently increases the levels of histone H3 Arg17 dimethylation and autophagy. CARM1 exerts transcriptional co-activator function through binding to the transcriptional activation domain of TFEB, whereas TFEB combines with the methyltransferase domain of CARM1 (Shin H. J. et al., 2016; Shin H. R. et al., 2016). An emerging phenomenon reveals that TFEB is an important upstream activator of some glycoproteins, which may be associated with increased levels of N-glycosylation and altered lysosomal protein activity in pancreatic ductal adenocarcinoma (Pan et al., 2014). However, it remains unclear on the specific mechanism responsible for TFEB regulating glycosylation and changes in the glycosylation levels of certain glycoproteins affecting the activity of TFEB.

As mentioned above, we briefly summarized those that are currently studied. It remains unclear how different pathways communicate with each other to regulate the activity of TFEB, and whether all of these different levels of modification ultimately affect lipid metabolism by regulating TFEB activity.

## The Function and Molecular Mechanism of TFEB Regulating Lipid Metabolism in Atherosclerosis

### The Role of TFEB in Lipid Homeostasis

After the transcriptional activity of TFEB itself is regulated by many other factors, it is activated into the nucleus and further regulates downstream targets to change intracellular metabolic changes, such as lipid metabolism (Settembre et al., 2013). The adaptive response of organisms to changes in nutritional status is related to major transcriptional and metabolic changes, one of which prominently observed during nutrient deprivation is the increase in lipid catabolism. During this period, lipid metabolism is regulated by many transcription factors, nuclear receptors, and non-coding RNA, which quickly adapt to the current environment to maintain the basic homeostasis and support the energy requirements (Li et al., 2017). TFEB is one of the transcription factors that efficaciously regulate lipid metabolism. It regulates not only the lipid transport processes such as lipid uptake and cholesterol efflux but also the lipophagy mediated by intracellular lysosomal lipolysis and neutral lipolysis adjusted by fatty acid oxidation-related proteins (Zechner et al., 2012). Microarray analysis identified numerous genes of lipid and fatty acid catabolic process, fatty acid-binding, and transport, fatty acid oxidation, sphingolipid catabolic process, steroid catabolic process et al., such the expression of lipid metabolic process genes are perturbed by TFEB overexpression (Settembre et al., 2013). Furthermore, TFEB has been found to also work cooperatively with transcription factor E3 (TFE3) in controlling whole-body metabolism, including lipid catabolism, energy metabolism, glucose homeostasis, and mitochondrial β-oxidation (Pastore et al., 2017). Accordingly, if the focus of basic research is turned to the transcriptional regulation of lipid metabolism by TFEB, it sheds light on the more thorough understanding of lipid trafficking pathways and homeostasis.

#### TFEB and Lipid Transporters

Transcription factor EB influences the expression of CD36, scavenger receptor class A (SR-A), ATP-binding cassette transporter A1 (ABCA1), and other proteins to regulate lipid transport. The level of these transporters, which is responsible for cholesterol uptake and efflux on the surface of macrophages, is dynamically changed to deal with the environment stimulation (Ye et al., 2019). The results have proved that hypericin-mediated sonodynamic therapy (HY-SDT) promotes the nuclear translocation of TFEB by generating reactive oxygen species (ROS) in macrophages, with a resultant increase in the level of macrophage cholesterol efflux and the expression of ABCA1 protein, and also inhibition at the mRNA levels of CD36 and SR-A. These findings indicate that TFEB is a momentous regulator that promotes lipid degradation and efflux of macrophages, and exhibits the potential therapeutic effect of inhibiting lipid uptake in response to atherogenic lipid stressors. Besides, this result also unveils that TFEB is a crucial regulator that promotes autophagy activation and lysosomal regeneration to ultimately reduce lipid accumulation in macrophages and ameliorate lipid overload in atherosclerotic plaques (Li X. et al., 2016).

#### TFEB and Lipid Degradation

When nutrients are lacking, lipid droplets are broken down to release energy. Maintaining the dynamic balance of lipid flux requires the synergistic regulation of autophagy-dependent lipolysis and neutral lipolysis (Zechner et al., 2017; Khawar et al., 2019). Autophagy-dependent lipolysis is the major way to adapt to environmental challenges, allowing removal of excess substrates and repair of damaged organelles (Maan et al., 2018). While neutral lipolysis is the key basis for lipid degradation, which provides necessary lipids for autophagic cell membranes (Tall, 2017; Schott et al., 2019). Additionally, neutral lipolysis may be a powerful effector of autophagy-dependent lipolysis as a result of making oxidative phosphorylation more allowable for mitochondrion.

Autophagy is a long-term evolutionary and reserved degradation system in cells that maintains the stability of the intracellular environment by degrading protein aggregates and damaged organelles, playing a central role in lipid metabolism (Martinez-Lopez and Singh, 2015). The lipid droplets accumulated in the cytoplasm are wrapped by a double-layer membrane structure to form autophagosomes, which then are fused with the lysosomes. Thereafter, the lipid droplets shuttle to the lysosome and are then degraded by acid lipase to produce free cholesterol and free fatty acids (Chen K. et al., 2017). Among them, the free cholesterol flows out of the cytoplasm through the mediation of transmembrane protein ABCA1 and is metabolized along with the blood circulation back to the liver to participate in the process of reverse cholesterol transport (Ouimet et al., 2019). Whereas free fatty acids are effluxed and re-uptake into mitochondria, where they undergo β oxidation to produce energy or ketone bodies for supporting cellular energy requirements. This selective autophagy process, also specifically known as lipophagy, is the lipid conversion of intracytoplasmic lipid droplets through the autophagy pathway, and the main way for lipid metabolism caused by long-term hunger in the body. As TFEB is a key regulatory factor for autophagy and lysosome, some compelling evidence suggests that TFEB modulates the degradation and efflux of intracellular lipids by regulating lipophagy-related genes after transferring from the cytoplasm to the nucleus (Thomes et al., 2019).

This is substantiated by studies demonstrating that TFEB inhibition abolishes lipophagy and makes for the decomposition of cellular lipid in anti-non-alcoholic fatty liver disease (Wang et al., 2019). Furthermore, in mouse models of obesity, suppression of TFEB, and Atg7 by liver-specific gene deletion facilitate liver steatosis and weight-gain (Yang et al., 2010). Helix-Loop-Helix-30 (HLH-30), the TFEB ortholog as transcriptional switches, couple autophagy and lysosomal lipolysis to nutritional changes through mediating the efficient lipid clearance in Caenorhabditis Elegans, resulting in additive effects with controlling fat storage (O’Rourke and Ruvkun, 2013; Franco-Juárez et al., 2018). In agreement with this, a similar conserved pathway is replicable in human cells (Ji et al., 2020) and murine models (Visvikis et al., 2014; Nakamura and Yoshimori, 2018), underscoring that HLH-30 or TFEB is conservative in linking autophagy to lipid homeostasis and lifespan. There are also reports for regulation by TFEB of peroxisome proliferator-activated receptor-γ coactivator-1α (PGC-1α) (Finck and Kelly, 2006) and peroxisome proliferator-activated receptor α (PPARα) (Tan H. W. S. et al., 2019), the main regulatory factors of lipid metabolism. Support for this concept is the finding that PGC-1α activation promoted by TFEB targets the downstream PPARα and controls lipid degradation (Evans et al., 2019). In macrophages infected by mycobacteria, PPARα agonists promote the transcription of TFEB, lipid catabolism, fatty acid β-oxidation, and inhibit lipid formation for antimycobacterial effect (Kim Y. S. et al., 2017), which emphasizes the interplay between autophagy and lipid metabolism at the transcriptional level. Recent studies show that TFEB participates in store-operated calcium entry-controlled lipid metabolism, inducing lipophagy, and mobilization of fatty acid from lipid droplets (Maus et al., 2017; Zhu et al., 2018). Besides, TFEB plays a part in lipid catabolism mediated by phosphatidylinositol-5-phosphate 4-kinases required for autophagosome-lysosome fusion (Lundquist et al., 2018). These reliable reports provide proof that TFEB is connected closely with lipophagy *via* the autophagy-lysosome pathway. But the key of lipophagy is sufficient autophagic flux and complete autophagy-lysosome form and fusion pathway to produce enough autophagy cycles for phagocytosing excess lipids.

Neutral lipolysis is the process in which cytoplasmic lipases such as the key cytoplasmic lipase, adipose triglyceride lipase (ATGL), and hormone-sensitive lipase (HSL) exert the hydrolytic effect by directly attaching to the surface of lipid droplets, and serving as another capital intracellular lipid degradation mode. Together with lipophagy, lipolysis constitutes the key to the regulation of lipid droplet catabolism (Kloska et al., 2020). Studies have shown that lipase-driven decomposition of lipid droplets has proved part of its process depends on autophagy. Since it was observed that ATGL and HSL both contain motifs that directly interact with LC3, it is speculated that the resulting up-regulation of hepatic lipid phagocytosis has a synergistic effect with lipolysis (Martinez-Lopez et al., 2016; Cui et al., 2020). Similarly, ATGL activity may also be an influential checkpoint for regulating lipid phagocytosis, and ATGL overexpression may increase liver fat phagocytosis *via* sirtuin-1 (Sathyanarayan et al., 2017). Therefore, lipophagy and lipolysis are closely related. As the role of TFEB in lipophagy is becoming more and more clear, it is also important to explore the role of TFEB in the lipolysis process. While there are few related reports at present and further investigation is needed.

In addition to the aforementioned approaches involving autophagy-related targets in regulating lipid metabolism, TFEB also binds directly to cholesterol 7α-hydroxylase (CYP7A1) promoter in human and mouse hepatocytes, which augments bile acid synthesis resulting in cholesterol catabolism and degradation. This mechanism may contribute to the prevention of hypercholesterolemia and metabolic disorders. Bile acid-induced fibroblast growth factor 19 mediates feedback inhibition of TFEB activity and nuclear translocation *via* activating the mTOR/ERK pathway and phosphorylating TFEB (Wang et al., 2020a).

#### TFEB and Bioactive Lipid Mediators

It is well known that lipids metabolism produces many small molecules, acting as bioactive lipid mediators to participate in cell signaling pathways and mediate numerous biological effects (Huang and Freter, 2015). Such key bioactive lipids include fatty acids, diacylglycerol, sphingolipids, ceramide, sphingosine, sphingosine-1-phosphate, and others. TFEB promotes fatty acid oxidation in mitochondria and peroxisomes and inhibits fatty acid biosynthesis. Strikingly, TFEB action is a result of the different fatty acid types and duration of exposure to exogenous fatty acids in murine cardiomyocytes (Trivedi et al., 2020). TFEB content is decreased in a concentration- and time-dependent manner by palmitate, the saturated fatty acid, rather than polyunsaturated fatty acids. Animal models show that a high-fat and high-sucrose diet induces a temporary decline in nuclear TFEB, concomitant with the molecular events that lipid deposition is induced by marked elevation of diacylglycerol and triacylglycerol. Early studies show that some lysosomal enzymes that regulate the degradation of sphingolipids and glycogen become direct targets of TFEB (Palmieri et al., 2011), implying the indirect regulation of TFEB on lipid mediators. In murine (C57BL/6) lungs, TFEB/lipophagy induction substantially reduces cigarette smoke-mediated intracellular-ceramide-accumulation to normal levels (Bodas et al., 2019).

### The Pathological Process of Atherosclerosis

Atherosclerosis is a chronic inflammation-related pathological process caused by various reasons (Yang M. Y. et al., 2018; Ray et al., 2019). The hallmarks of atherosclerosis are lipid accumulation and chronic inflammation of that arterial wall. Under the action of various risk factors such as smoking, obesity, hypertension, and dyslipidemia, the oxidized low-density lipoprotein (ox-LDL) causes endothelial damage and dysfunction ascribed to the state of hyperlipidemia (Negre-Salvayre et al., 2020), leading to increased levels of adhesion factors and chemokines. These cytokines promote the monocytes to be converted into macrophages to devour lipids through the adhesion at the injury site of arteries and migration to the subendothelial layer. The degree of lipid accumulation in macrophages represents the balance between extracellular lipid uptake and lipid reverse transport. When the influx and esterification of intracellular lipids are greater than the outflow, the accumulation of lipids occurs, and the excess lipids are stored in the lipid droplets. Therefore, macrophage-derived foam cells promote the formation of lipid striae and become the kernel of plaques (Guerrini and Gennaro, 2019). In parallel, smooth muscle cells of the medial membrane migrate into the subendothelial layer and gradually accumulate to form a new fibrous cap of atherosclerotic plaque, which enlarges the plaque to rupture injury. If the body is unable to promptly clear the apoptotic foam cells, a thrombus, and an inflammatory necrosis core is formed, eventually leading to acute coronary syndrome (Paone et al., 2019).

### The Importance of Lipid Homeostasis for Atherosclerosis

Many factors promote atherosclerosis, among which lipid metabolism disorder is a conspicuous pathological factor contributing to the formation and progression of atherosclerosis. It is generally believed that foam cells are the typical features of early atherosclerosis (Maguire et al., 2019). The main lipid types in these foam cells are free cholesterol and cholesterol esters, which are stored in the form of lipid droplets. In mammals, lipid droplets play a standout role in intracellular lipid balance. Damaged lipophagy causes lipid accumulation and deranges lipid metabolism, which aggravates atherosclerosis progress. Therefore, reducing lipid uptake, or mobilizing the release of cholesterol in the cytoplasm from lipid droplets, and enhancing lipid catabolism and efflux are principal for understanding the physiological mechanisms regulating lipid homeostasis and reversing lipid content of plaques (Yang M. et al., 2018). It provides a prominent theoretical basis for us to develop effective drug targets to reduce the formation of foam cells constituting an attractive method for the prevention and treatment of atherosclerosis. Over the past years, with the study of lipid homeostasis in atherosclerosis showing the situation of the explosive growth, the regulatory role of TFEB has also been gradually explored.

### TFEB, Lipid Homeostasis, and Atherosclerosis

Transcription factor EB is ubiquitously expressed in multifarious cell lines, performing the functions of regulating lipid homeostasis in many diseases. For the reason that lipid metabolism is closely related to atherosclerosis, it is hypothesized that TFEB also regulates lipid metabolism in atherosclerosis. Therefore, the transcription function of TFEB has attracted the attention of scientific researchers in the atherosclerosis field.

Many studies have confirmed that TFEB inhibits the progression of atherosclerosis mainly by promoting lipid degradation. During the development of atherosclerosis, lipids are catabolized in lysosomes. Adequate lipid catabolism has the effect of delaying the progression of atherosclerosis. However, excessive lipid accumulation in lysosomes may cause progressive dysregulation of lysosomes and the autophagy function. Therefore, lysosomes show the characteristics of larger volume and decreased protein degradation ability, which aggravates the deterioration of the plaque microenvironment and accelerates the plaque rupture. It is reported that the promotion of macrophage autophagy metabolizes lipid droplets into free fatty acids, which is of stupendous significance in promoting lipid efflux in atherosclerosis.

As a regulator of the autophagy-lysosome pathway, TFEB up-regulates the expression of 2/3 autophagy-lysosome-related genes. Studies have found that TFEB overexpression promotes the synthesis of lysosomes and related enzymes to rescue damaged lysosomes. Enhancing autophagy promotes the clearance of damaged proteins, alleviates lipid-induced macrophage lysosomal dysfunction, and provokes lipid metabolism (Emanuel et al., 2014). In vascular smooth muscle cells, proteomic analysis, and database searching identified an appealing protein stearoyl-CoA desaturase 1 (SCD1). Overexpressed SCD1 has a promotional effect on the TFEB nuclear translocation and its reporter activity, mediating lipophagy for inhibiting foam cell formation (Pi et al., 2019). Analogously, in APOE^–/–^ mice with macrophage depletion, sorting nexin 10 deficiency enhances TFEB nuclear translocation exerting the effect of promoting activity of lysosomal acid lipase and mitochondrial fatty acid oxidation, coinciding with interrupting the internalization of CD36 to prevent the uptake of lipids, thereby inhibiting foam cell formation (You et al., 2020). TFEB overexpression also inhibits atherosclerosis plaques from the aspect of promoting lipid transport. Li et al. demonstrated that TFEB was shown to promote cholesterol and fatty acid efflux by enhancing the expression of ABCA1 while inhibiting lipid uptake by suppression of CD36 expression (Li X. et al., 2016).

## Application of TFEB as a Target in Disease Treatment

Based on the understanding of the above regulatory mechanisms, the function of TFEB in lipid metabolism is increasingly clear. Recent research effort has devoted into developing drugs targeting the regulation of TFEB to treat several diseases in which a defective lipid and energy metabolism is a key contributor to the disease pathogenesis. Indeed, some gratifying progress has been made. Three lead compounds as TFEB efficacious agonists have been identified through a nanotechnology-enabled high-throughput screening strategy, embracing digoxin, ikarugamycin, and alexidine dihydrochloride, which belong to a small cohort of clinically approved drugs, the marine-derived natural product, and synthetic small molecules, respectively. These compounds engage TFEB activation pathways *via* three distinct mobilizations of Ca2^+^-dependent mechanisms, which improve lipid metabolism with relieviated steatosis in C57BL/6 mice fed a high-fat diet (Wang et al., 2017). Procyanidin B2, a naturally occurring phenolic compound, holds the promise to be developed as a novel drug for the treatment of non-alcoholic fatty liver disease, as its function in modulating lysosomal pathway and redox state mediated by TFEB to attenuate free fatty acids-induced hepatic steatosis (Su et al., 2018). The cholesterol-lowering agent ezetimibe is widely used for hypercholesterolemia. In mice treated with a methionine- and choline-deficient diet, ezetimibe attenuates lipid accumulation and steatohepatitis by autophagy activation through phosphorylating AMPK and promoting TFEB nuclear translocation (Kim S. H. et al., 2017). As a natural product, formononetin has been shown to protect against hepatosteatosis by promoting TFEB-mediated lipophagy (Wang et al., 2019). A very recent study reveals that dihydromyricetin initiates autophagic cutaneous squamous cell carcinoma (CSCC) cell death through inducing TFEB (S142) dephosphorylation and increasing the activity of TFEB reporter, which is conducive to CSCC chemotherapy (Tan M. et al., 2019).

As noted above, some small-molecule substances regulate the activity of TFEB in atherosclerosis. Disaccharide trehalose is shown to exhibit atheroprotective effects. As a beneficial inducer of TFEB, disaccharide trehalose orchestrates the functional autophagy-lysosome system for lipid degradation in the atherosclerotic plaque (Sergin et al., 2017; Evans et al., 2018). HY-SDT was found to be effective in improving the progression of atherosclerosis through triggering TFEB nuclear translocation and promoting lipid degradation and efflux, decreasing the lipid content (Li X. et al., 2016). The research on TFEB linked with atherosclerosis is still in the early stage. Current research is only limited to the discovery of several small molecular substances that might be used as TFEB agonists to inhibit plaques in mice. Studies have proved that the overexpression of TFEB reduces the samples of human atherosclerotic plaques (Sergin et al., 2017), but no agent targeting TFEB has been found to treat atherosclerosis patients. Nevertheless, these findings are encouraging in terms of potential therapeutic application, as an interventional treatment after disease onset would be a valuable advantage.

## Concluding Remarks

As an important transcription factor that regulates cell homeostasis and body pathogenesis, TFEB has attracted tremendous attention in various fields credited with its transcription function system. The unambiguous mechanism of its activity remains to be elucidated. Although post-translational modification is considered to be the main way to regulate TFEB activity and intracellular localization, currently only phosphorylation modification has received thorough research, whereas the other powerful regulation machinery, such as acetylation, SUMOylation, and ubiquitination, need to be explored. It is also necessary to determine the particular sites of post-translational modifications regulating the TFEB function, which is also challenging. Another vital issue to be solved is how TFEB is transferred to the cytoplasm once it enters the nucleus, and how TFEB export from the nucleus is regulated and whether it exerts additional roles.

Under stress conditions, TFEB undergoes nuclear translocation and binds to the corresponding DNA fragments to regulate the expression of proteins related to the autophagy-lysosome pathway. The information on environmental cues is transmitted to the nucleus to trigger a transcription response. Particularly, TFEB links different organelles such as lysosomes and the nucleus to enhance overall metabolism. There have been many data proving the importance of TFEB in all levels of the body, but it is still necessary to clarify the idiographic cellular function mediated by TFEB under different research background such as different types of cells or tissue and the nature of external stress conditions.

Due to its involvement in the intracellular clearance pathway, TFEB has been shown as an essential factor for lipid degradation and has made preliminary progress in regulating lipid transporters and lipid mediators. However, more high-quality, in-depth study of its definite mechanisms are still needed, and the comprehensive regulatory network of TFEB in lipid metabolism awaits perfection, considering the limited understanding of TFEB in lipid synthesis, mobilization, esterification, transport. Lipid disorders and inflammation are the two main characteristics of atherosclerosis. The regulation mechanism of TFEB in immune inflammation has been roughly determined (Brady et al., 2018b), but the regulation of lipid homeostasis of TFEB in atherosclerosis needs to be fully studied.

With the continuous deepening of molecular biology research, various mechanisms regulating TFEB and targeted drugs have been understood to a certain extent. However, the current research is only limited to the transcription level and the protein level and mainly focuses on the dephosphorylation of TFEB which gets into the nucleus and increases its activity. There are no intervention drugs to be able to modulate the acetylation of TFEB protein. Undoubtedly, it is a demand to explore effective drugs targeting TFEB continuously. In conclusion, the transformation from TFEB basic research into clinical applications is crucial. An in-depth exploration of the explicit mode of action of TFEB on lipid metabolism and the dialogue mechanism with other signaling pathways will improve our understanding on the regulation mechanism of lipid metabolism in atherosclerosis and provide new perspectives and strategies for the prevention and treatment of related metabolic diseases.

## Author Contributions

ML, HL, and LY designed and wrote the manuscript. All authors edited the manuscript.

## Conflict of Interest

The authors declare that the research was conducted in the absence of any commercial or financial relationships that could be construed as a potential conflict of interest.

## Abbreviations

ABCA1: ATP-binding cassette transporter A1
ACAT1: acetyl-coenzyme A acyltransferase1
AD: activation domain
AKT: protein kinase B
AMPK: adenosine monophosphate-activated protein kinase
ATGL: adipose triglyceride lipase
bHLH: basic helix-loop-helix
bHLHZIP: basic helix-loop-helix leucine-zipper
CARM1: co-activator-associated arginine methyltransferase 1
ChIP: chromatin immunoprecipitation
CLEAR: coordinated lysosomal expression and regulation
CSCC: cutaneous squamous cell carcinoma
CYP7A1: cholesterol 7 α -hydroxylase
DAG: diglyceride
DNA: deoxyribonucleic acid
ERK: extracellular signal-regulated kinase
G: glycerol
GCN5: general control non-repressed protein 5
Gin: glutamine
GSK-3β: glycogen synthase kinase-3 β
HDAC2: histone deacetylase 2
HDAC6: histone deacetylase 6
HLH-30: Helix-Loop-Helix-30
HSL: hormone-sensitive lipase
HY-SDT: hypericin-mediated sonodynamic therapy
JAK2: Janus kinase 2
LncRNA: long non-coding RNA
LZ: leucine-zipper
MAG: monoacylglycerol
MAGL: monoacylglycerol lipase
METTL3: methyltransferase like 3
miR-128: microRNA-128
MiT/TFE: microphthalmia/transcription factor E
mTOR: mammalian target of rapamycin
mTORC1: mammalian target of rapamycin complex 1
ox-LDL: oxidized low-density lipoprotein
PGC-1α: peroxisome proliferator-activated receptor- γ coactivator-1 α
PKC: protein kinase C
PPARα: peroxisome proliferator-activated receptor α
Pro: proline
ROS: reactive oxygen species
SAHA: suberoylanilide hydroxamic acid
SCD1: stearoyl-CoA desaturase 1
SR-A: scavenger receptor class A
SUMO: small ubiquitin-like modifier
TAG: triglyceride
TFE3: transcription factor E3
TFEB: transcription factor EB
YWHA 14-3-3: phospho-serine/phospho-threonine binding protein.
